# Validation of diffusion and exchange imaging biomarkers via simultaneous real-time NMR and optical microscopy

**DOI:** 10.64898/2025.12.30.697003

**Published:** 2025-12-30

**Authors:** Rea Ravin, Nathan H. Williamson, Teddy X. Cai, Peter J. Basser

**Affiliations:** 1 *Eunice Kennedy Shriver* National Institute of Child Health and Human Development, National Institutes of Health, Bethesda, MD 20892, USA; 2 Celoptics, Rockville, MD 20850, USA; 3 Military Traumatic Brain Injury Initiative (MTBI^2^), Bethesda, MD 20814, USA; 4 Uniformed Services University of the Health Sciences (USU), Bethesda, Maryland 20814, USA; 5 The Henry M. Jackson Foundation for the Advancement of Military Medicine Inc. (HJF), Bethesda, Maryland 20817, USA

**Keywords:** Diffusion exchange Spectroscopy (DEXSY), validation, multimodal, extracellular space (ECS), low-field high-gradient NMR, transmembrane water exchange, cell volume regulation, spreading depolarization (SD)

## Abstract

Diffusion MRI can reflect features of tissue microstructure and homeostasis, but direct validation in living neural tissue remains challenging. Here we combine optical microscopy and NMR for real-time recording on viable *ex vivo* neural tissue during environmental perturbations. Simultaneous high-temporal-resolution NMR and optical microscopy are used to monitor apparent diffusion coefficient (ADC), apparent exchange rate (AXR), intrinsic optical signal (IOS), and intracellular calcium in *ex vivo* neonatal mouse spinal cord during osmotic and ionic perturbations. We find that ADC correlates strongly with IOS, while AXR decreases with depolarization. ADC and AXR are sensitive to distinct features of cellular swelling, supporting their complementary roles in probing tissue viability and function.

Diffusion MRI can probe features of cellular structure and homeostasis in living tissues such as the central nervous system (CNS), but its metrics often lack direct cross-validation.^1^ Most validation efforts have combined MRI with microscopy, but the approach and findings are not easily generalized due to their use of fixed *ex vivo* tissue,^2–6^ where cellular function is halted and membrane permeability and compartmental volumes are altered.^7,8^ While *in vivo* diffusion MRI has the potential to elucidate some physiological processes,^9–14^ it suffers from low sensitivity, slow acquisition, confounding effects from blood flow and respiration,^15,16^ and challenges when combined with other modalities.^17–19^ We show that real-time NMR measurements with viable *ex vivo* tissue can overcome these limitations by preserving physiological function while enabling exquisite experimental control and relative ease to combine virtually any type of optical microscopy.

NMR can characterize diffusion and the steady-state exchange of water noninvasively.^20,21^ Magnetic field gradients can be used to encode molecular displacements along the direction of the gradient and to measure the apparent diffusion coefficient (ADC). Differences in diffusion properties within the intracellular and extracellular environments can be observed on timescales where water on average has felt the effect of plasma membranes but has not yet permeated them. On these timescales, water inside cells is less mobile because it is bound and restricted by membranes, whereas water outside is more mobile because it can diffuse through a tortuous but connected extracellular space (ECS). In this way the ADC may be used as a proxy for cell volume, but not necessarily quantitatively due to structural heterogeneity. In particular, the heterogeneity of cell sizes and orientations of cellular processes can lead to water in some intracellular domains appearing more mobile than others. Exchange between environments where water mobility differs can be measured by encoding the same water at two instances separated by a mixing time where the water can communicate between environments.^22,23^ In this way, the apparent exchange rate constant (AXR) is sensitive to membrane permeability,^24,25^ however not quantitatively due to the heterogeneity in cell size, and thus cell surface-to-volume ratios, and not specifically due to the sensitivity to other exchange pathways such as diffusion-mediated, geometric exchange between domains inside the cell.^26–28^

Steady-state water exchange is a fundamental aspect of cellular homeostasis.^29^ Water exchange occurs by passive diffusion^27,30^ and, potentially, active transport processes.^31–33^ NMR provides one of the only direct means to measure steady-state water exchange, but measurements are time consuming because they require repeated scans with different encodings. Validation of exchange metrics is especially challenging since exchange and structure are intertwined and there is no method for direct comparison. This necessitates approaches that characterize as many inputs as possible simultaneously and interpret them within the physiological context of the living system. In CNS gray matter, exchange across densely packed neuronal and glial processes is expected to be rapid due to their high surface-to-volume ratios^34,35^ and potentially high membrane permeability.^36,37^ Capturing these rapid dynamics demands methods with both the spatial resolution to probe fine structural compartments and the temporal resolution to follow fast exchange processes—capabilities achieved by the real-time NMR methods developed here.

In this study, we combine new real-time, high-temporal-resolution NMR with high-spatial and temporal resolution optical microscopy of intrinsic optical signals (IOS), and intracellular calcium ([Ca^2+^]_i_) imaging to validate the sensitivity of the apparent diffusion coefficient (ADC) and apparent exchange rate (AXR) to changes in cellular structure and function. Using viable *ex vivo* neonatal mouse spinal cords with a solenoid RF coil provides a high filling factor and roughly a 10-fold SNR improvement over previous flat coil designs.^38^ To accommodate an inverted microscope positioned beside the NMR magnet, an objective inverter was used. An immersion objective minimized fluctuations from flowing media and provided a high numerical aperture. We use a single-sided permanent magnet which provides access to the sample from above and provides an extremely strong g=15.3T/m static gradient (SG) for measuring ADCs on sub-millisecond diffusion timescales, setting the higher limit for measurable AXRs at 1000 s^−1^, an order of magnitude higher than can be measured with standard pulsed field gradient (PFG) approaches. During osmotic pressure (sucrose) and ionic (KCl) perturbations, we find that ADC correlates closely with IOS, suggesting similar biophysical origins—namely, cell shrinking and swelling. Meanwhile, building on findings from our previous work,^29^ AXR remains relatively stable during periods of increased osmolarity but drops when [Ca^2+^]_i_ peaks during KCl addition, suggesting that it reflects loss of tissue homeostasis during spreading depolarization.

## Results

### real-time diffusion and exchange measurement and validation.

We first develop quantitative real-time NMR methods to characterize the homeostatic state and are fast enough to observe changes induced by osmotic (sucrose) and ionic (KCl) perturbations to the media bathing the spinal cord. Concentration gradients across the *≈* 0.7 mm sample radius are expected to equilibrate on a timescale of *≈* 1 minute for water,^8^ slightly longer for K^+^, and roughly four times longer for sucrose based on their self-diffusion coefficients.^39,40^ Mechanisms such as volume regulation and K^+^ buffering act to preserve homeostasis during perturbations, but at times these are not enough. Excess extracellular K^+^ triggers spreading depolarization (SD)—an “all-or-none” cascade of neuronal firing and K^+^ release propagating at millimeters per minute.^41^ SD occurs across a range of pathologies, including migraine aura, traumatic brain injury, and stroke.^42^ Bath application of 50 mM KCl is expected to evoke SD at the cord surface, spreading to the center within a minute without recovery, causing [Ca^2+^]_i_ elevation, IOS change, and ADC and AXR reduction from cellular swelling and extracellular shrinkage.^10,27,41,43,44^ To better match those dynamics—and to narrow the gap with the temporal resolution of microscopy—we developed a protocol which takes 40 seconds total per ADC and AXR measurement. This was achieved by understanding and isolating the experimental weighting parameter needed for the measurement.

The ADC measurement is based on SG spin echo (SG SE) diffusion encoding where the diffusion weighting (DW) is summarized by $b=2/3\gamma^{2}g^{2}\tau^{3}$ with gyromagnetic ratio γ and diffusion encoding time τ in the equation $S(b)=S_{0}\;\text{exp}(-b\text{ADC})$. With two free parameters, the analytical expression for finding the (1-dimensional) ADC with two signals S(1) and S(2) acquired at b(1) near zero and b(2) near 1*/*ADC is

(1) $$\text{ADC}=-\frac{1}{b\left(1\right)-b\left(2\right)}\text{ln}\frac{S\left(2\right)}{S\left(1\right)},$$

as is well-known in the field of diffusion MRI.^45^

The AXR measurement is based on the SG diffusion exchange spectroscopy (DEXSY) experiment which applies two diffusion encodings ($b_{1}$ and $b_{2}$) separated by a mixing time $\left(t_{m}\right)$.^8,22^ Early DEXSY implementations required hours to days per dataset because of extensive phase cycling and dense sampling of $b_{1}$ and $b_{2}$ values.^8,22,46,47^ Later methods such as filter exchange spectroscopy and imaging (FEXSY, FEXI) reduced acquisition time by optimizing signal sampling.^23,48–50^ Further improvements came from understanding how to isolate diffusion, exchange, and relaxation weightings.^51–57^

For two-site, barrier-limited exchange—where water molecules traverse the intracellular space (environment i) many times before crossing to the extracellular space (o)—exchange-weighted DEXSY signals attenuate with the apparent exchange rate (AXR) and longitudinal relaxation rate $\left(R_{1}\right)$ according to

(2) $$S\left(t_{\text{m}}\right)=\left(S_{0}\text{exp}\left(-t_{\text{m}}\text{AXR}\right)+B\right)\text{exp}\left(-R_{1}t_{m}\right).$$

In this case, $\text{AXR}=k=k_{io}+k_{oi}$, the sum of the rate constants for exchange between the two environments. The diffusion exchange ratio (DEXR) method uses prior knowledge of $R_{1}$ to reduce Eq. 2 to a three-parameter model.^53,57^ This allows AXR estimation from three relaxation-adjusted signals—acquired at short $\left(t_{m\text{,short}}\ll 1/\text{AXR}\right)$, intermediate $\left(t_{m,\text{int}}\approx 1/\text{AXR}\right)$, and long $\left(t_{m,\text{long}}\gg 1/\text{AXR}\right)$ mixing times via

(3) $$\text{AXR}=-\frac{1}{t_{m,\text{int}}}\text{ln}\left(1-\frac{S_{\text{short}}-S_{\text{int}}}{S_{\text{short}}-S_{\text{long}}}\right).$$

Both SE and DEXSY MR sequences were repeated four times per signal to cover the phase cycle steps which add together desired signals and subtract unwanted ones,^58,59^ still with a substantial improvement in efficiency compared to earlier static-gradient DEXSY implementations.^8,47^ With a 2 second repetition time, this leads to 16 seconds for an ADC measurement and 24 second for an AXR measurement. These sequences acquire signals using a Carr–Purcell–Meiboom–Gill (CPMG) echo train,^60–62^ which simultaneously yields DW and transverse relaxation rates $\left(R_{2}\right)$. While this ultra-fast approach cannot identify and characterize multiple exchanging and non-exchanging compartments, it also obviates the assumptions and complexity of models in which at least one of those compartments is restricted.

The 3-point AXR measurement has never been used before. We first validated it using both numerical simulations and subsampled DEXSY data.^29^ Simulations of two-site exchange showed quantitative accuracy for true rate constants $\left(k\right)$ between roughly 20 and 150 s^−1^ (Fig. S1). The range of accuracy could be tuned by shifting the mixing time values (Fig. S2). In data subsampled from oxygen and glucose deprivation (OGD) experiments on *ex vivo* neonatal mouse spinal cords, the method remained quantitative under normal conditions (AXR *≈* 140 s^−1^) but overestimated exchange after OGD, when AXR dropped to *≈* 40 s^−1^ (Fig. S3). This overestimation likely reflects deviations from two-site barrier-limited exchange.^59,63,64^

### Intrinsic Optical Signal: Comparison of reflected and transmitted light.

On the microscopy side, we focused on intrinsic optical signal (IOS) imaging because, while the mechanism is completely different and relies on tissue light scattering or transmittance changes, like diffusion MRI, it is sensitive to cellular-scale microstructural changes averaged over many cells in *ex vivo* CNS tissue.^65–73^ Although IOS is relatively simple to acquire, it has not previously been performed simultaneously with NMR.

IOS contrast depends on the angle between the light source and the objective.^67^ The single-sided magnet is sitting below the stage in close proximity to the stage and chamber, making it difficult to orient the light source below the sample. For this reason, we decided to use a 90° configuration, in which the light source and objective are oriented perpendicularly. Then, the detected light primarily arises from reflection and scattering within the sample. In contrast, magnets with an internal bore could more easily support a 180° configuration, where transmitted rather than reflected light reaches the detector. Because transmittance is expected to vary inversely with reflectance during microstructural changes such as cell swelling or shrinkage,^67^ we compared both geometries (IOS_90_ and IOS_180_) in separate experiments using the same system, but the latter performed without NMR.

**Fig. 1.**
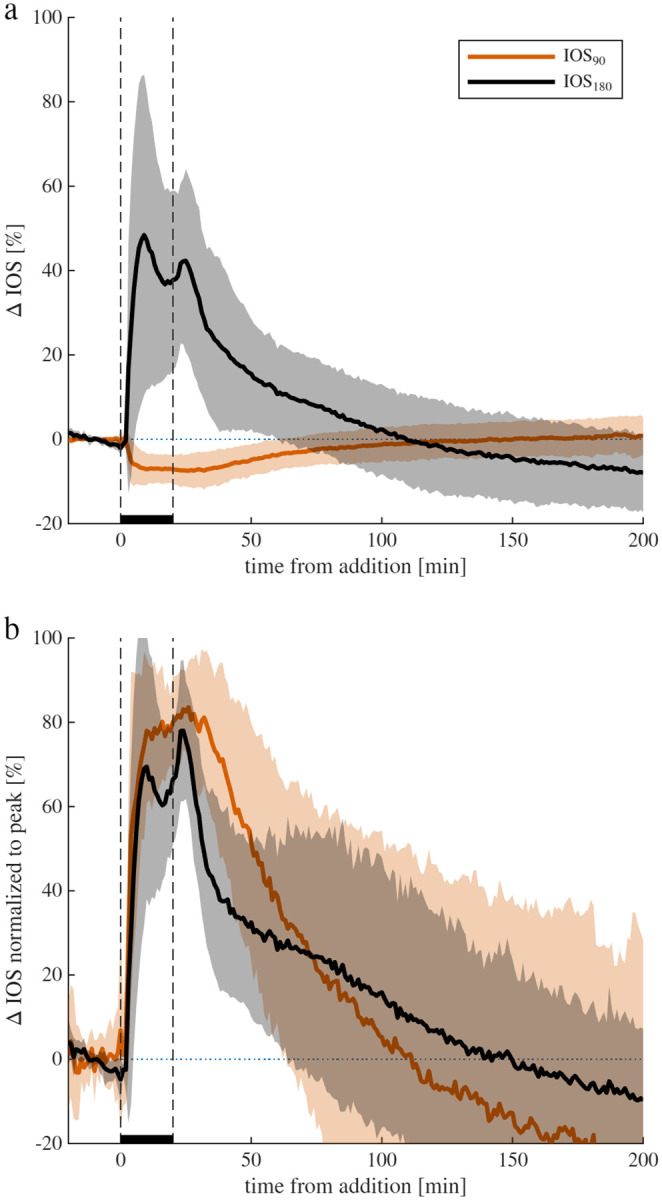
Comparison of 90° and 180° configurations. a) Percent change in IOS from baseline (mean ± standard deviation) for reflected light (IOS_90_, n = 13) and transmitted light (IOS_180_, n = 9) setups during exposure to 50 mM KCl at t=0 and washout after 20 min. b) Normalized signals scaled to the minimum (IOS_90_) or maximum (IOS_180_) values.

Following the addition of 50 mM KCl for 20 minutes and subsequent washout, IOS_90_ decreased while IOS_180_ increased (Fig. 1a) as expected from previous studies.^67^ When normalized to their peaks the signals were highly similar (Fig. 1b), consistent with prior findings that both reflected and transmitted light track cellular swelling.^67^ For compatibility with the NMR setup, subsequent experiments used only IOS_90_ (hereafter referred to simply as IOS).

### Simultaneous real-time NMR and microscopy demonstration.

Simultaneous microscopy and NMR were then initiated with real-time recordings. Spinal cords were bolusinjected with fluorescent indicator Rhod-3 AM for [Ca^2+^]_i_ imaging, and baseline NMR recordings were performed with the standard longer DEXR, $T_{1}$, and diffusion measurements. This served as a quality control on the viability of the sample, as AXR provides an absolute value that can be compared between samples.^29^ Samples for which AXR started too low (below 100 s^−1^) were discarded because they were found previously to run down and not recover.^29^

**Fig. 2.**
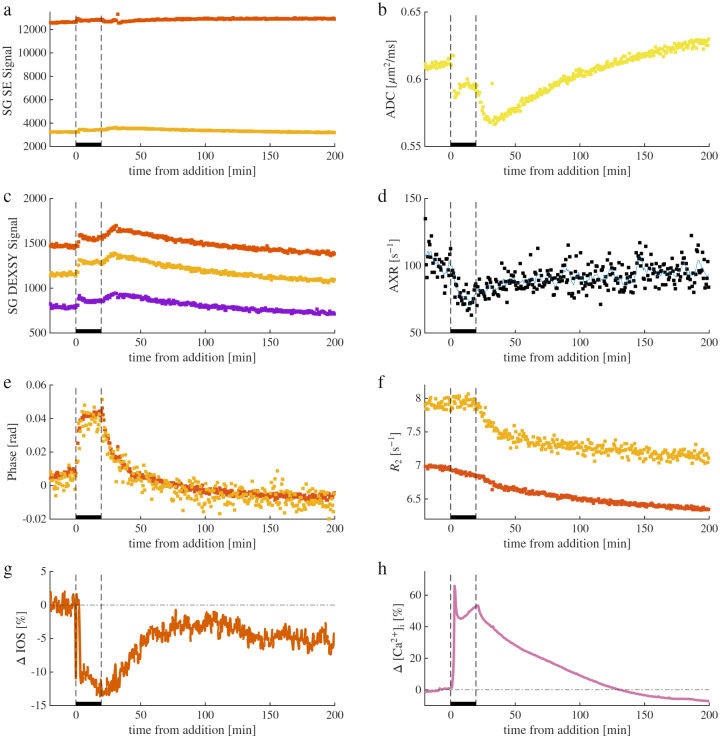
Example real-time NMR and microscopy recording during an experiment involving 50 mM KCl addition and washout. real-time recordings of two (b=0.025 and $2.25\;\text{ms}/\mu {\text{m}}^{2}$, red and orange dots, respectively) raw spin echo diffusion signals (a), processed into one ADC measurement using Eq. 1 (b), and three ($t_{m}=0.02$, 10, and 160 ms, red, orange, and blue dots, respectively) raw exchange-weighted DEXSY signals (c), processed into one AXR measurement using Eq. 3 (d). In (d), a 6-point moving average of AXR is also shown (solid blue line). e,f) Mean echo phase (e) and $R_{2}$ (f) recorded using the spin echo sequence with b=0.025 and $2.25\;\text{ms}/\mu {\text{m}}^{2}$ (red and orange dots, respectively). IOS images (g) and fluorescence images of [Ca^2+^]_i_ from the fluorescent indicator, Rhod-3 AM (h) are acquired simultaneously with the NMR, and the percent change in image intensity from a region of interest (ROI) is displayed.

Sample recordings from a 50 mM KCl perturbation experiment are shown in Fig. 2. Raw NMR signals from two SG SE and three SG DEXSY acquisitions were processed into ADC and AXR time-series using Eqs. 1 and 3, respectively (Fig. 2a–d). Because baseline ADC values vary across samples, subsequent figures report percent changes rather than absolute values. In contrast, baseline AXR values show little variation between samples, consistent with the interpretation that absolute AXR reflects tissue viability.^29^

Each NMR signal represents the summed echoes of the CPMG acquisition block. From these, we also extract phase and transverse relaxation rates $\left(R_{2}\right)$ (Fig. 2e–i). Phase reflects ionic composition and indicates equilibration between the bathing medium and tissue. Following KCl addition, phase equilibrates within *≈* 1 min, consistent with chamber turnover, whereas washout occurs more slowly (*≈* 10 min), perhaps limited by K^+^ transport out of cells. The absence of b-dependence in phase confirms negligible coherent flow along the gradient direction.

$R_{2}$ reflects rotational mobility,^74^ and increases with decreasing mobility.^75^ Here, nearly all the signal comes from protons on water,^8^ and $R_{2}$ varies due to chemical and physical interactions between molecules and surfaces that affect water’s molecular tumbling rate. $R_{2}$ and ADC are expected to be negatively correlated for diffusion in porous media because both are sensitive to the size of restrictions.^76^ We find that $R_{2}$ values increase with diffusion weighting, consistent with faster relaxation in more restricted environments. Their time courses differ: $R_{2}$ declines steadily after KCl addition and washout, whereas diffusion-weighted $R_{2}$ decreases only during washout. Although these trends are noteworthy, we focus below on the diffusion (ADC) and exchange (AXR) metrics. NMR metrics are acquired simultaneously with IOS and [Ca^2+^]_i_ (Fig. 2 g and h) (Fig. 2g,h). During the wash, [Ca^2+^]_i_ drops below baseline, likely due to the calcium indicator slowly diffusing out of the region of interest and not indicating [Ca^2+^]_i_ being lower than at baseline. Further trends will be discussed below. An example recording from a 100 mM sucrose perturbation experiment is shown in Fig. S4.

### Simultaneous real-time NMR and microscopy shows cellular shrinkage caused by adding an osmolyte and cellular swelling during depolarization from 50 mM KCl.

**Fig. 3.**
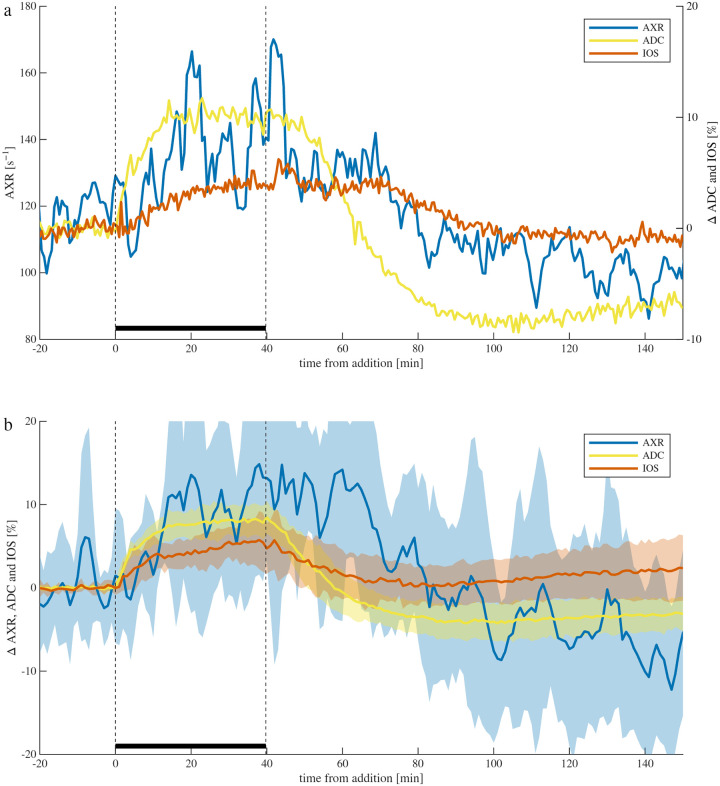
Simultaneous real-time NMR and microscopy shows cellular shrinkage caused by adding an osmolyte. a) A representative sample and b) means (lines) and standard deviations (shaded bands) across n=8 samples of real-time AXR (6-point moving average), ADC, and IOS recording during experiments involving 100 mM sucrose being added for 40 minutes and then washed away.

In the first set of experiments, ADC and AXR from NMR were acquired simultaneously with IOS from microscopy during perturbations with 100 mM sucrose over 40 minutes (Fig. 3). Sucrose is an osmolyte that does not penetrate across the plasma membrane. The 100 mOsm osmotic pressure pulls water from the intracellular space (ICS) to the extracellular space (ECS), shrinking the cells until the intracellular pressure matches the extracellular pressure. This effect is seen by the increase in ADC and IOS. Volume regulation by partitioning additional ions and metabolites to the ICS subdues the effect.^77^ AXR increases significantly $\left(p<0.001\right)$, but the effect is small (8.2 ± 23.2%). Results of previous studies involving Na^+^*/*K^+^–ATPase inhibition and energy failure showed AXR remaining stable and dropping when homeostasis was lost, suggesting that provides a proxy for homeostasis.^29^ Digging deeper into this mechanism, we found that water does not actively cycle with ions in this system. We did find that AXR is sensitive to tonicity and proposed a multisite exchange mechanism in which AXR increases with ECS fraction due to biasing of the measurement from slower geometric exchange to faster transmembrane exchange.^27,28^ The small effect of 100 mOsm sucrose on AXR and ADC has been shown previously^27^ and is consistent with homeostasis being maintained but a slight increase in ECS fraction.

During the wash back to normal media, ADC and IOS recovered, although with slightly different timecourses. ADC decreased faster than IOS and overshot the baseline. The recovery from ADC overshoot was incomplete, with ADC values between 100 to 200 minutes after addition being 2.8 ± 1.6% lower than baseline. The overshoot, indicative of cellular swelling, could be due to cells adjusting to a new steady state during the sucrose addition, but then having a higher intracellular osmolarity relative to the ECS, pulling water into the cells during the wash. AXR decreased significantly during the wash $\left(p<0.001\right)$, with AXR from 100 to 200 minutes after addition being 7 ± 19% lower than baseline.

Out of the eight experiments, from the data acquired between t=0 to 80 minutes after osmolyte addition, the correlation between AXR and ADC was only significant $\left(p<0.01\right)$ in two cases. The correlation between AXR and IOS was only significant in one case. The correlation between ADC and IOS was significant in six cases, and the correlation coefficient $\left(cc\right)$ was 0.78 ± 0.10 for those cases.

**Fig. 4.**
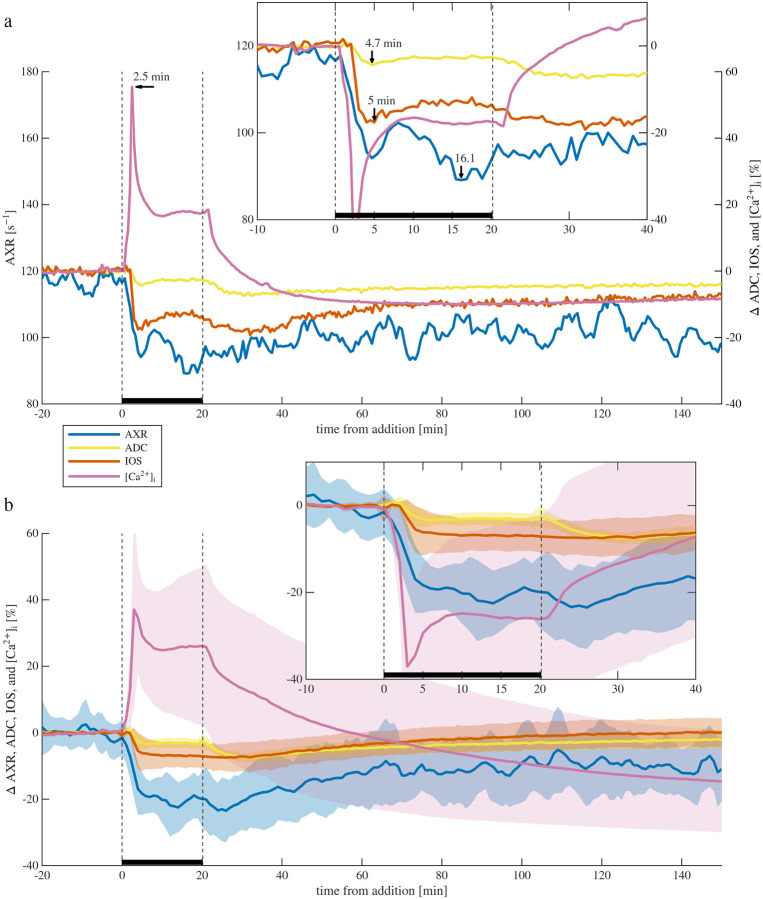
Simultaneous real-time NMR and microscopy reveal cellular swelling during depolarization from 50 mM KCl. a) Representative sample and b) mean ± SD of AXR (6-pt moving average), ADC, IOS, and [Ca^2+^]_i_ during 20 min KCl exposure and washout (n=12, three of which included [Ca^2+^]_i_ imaging). Insets show zoomed regions with [Ca^2+^]_i_ inverted for comparison. Arrows in (a) mark signal minima and maxima.

In the next set of experiments, similar data was acquired during 20 minute perturbations with 50 mM KCl, but with [Ca^2+^]_i_ signal being additionally acquired (Fig. 4). While 50 mM KCl dissociates into 100 mM of ions, the tonicity is lower than 100 mOsm because of the membrane permeability. Instead of acting as an osmolyte, 50 mM KCl is expected to induce spreading depolarization, causing cellular swelling and ECS shrinkage.^41^ Spreading depolarization is known to be accompanied by increased [Ca^2+^]_i_ which may be due to reversal of the Na^+^*/*Ca^2+^ exchanger,^78^ activation of calcium channels,^79^ mitochondrial depolarization and activation of the permeability transition pore,^44^ transport from endoplasmic reticulum,^80^ and transport through glial gap junctions.^43^ While [Ca^2+^]_i_ imaging can be used to monitor SD,^43^ the effect is indirect since SD can occur even when calcium is removed.^41,81^ IOS is also used to monitor SD.^43,44,81^ Interestingly, calcium waves have been found to occur before changes in IOS.^81^ Since the KCl is bath-applied, the sample is expected to remain depolarized until the wash. Depolarization and loss of homeostasis is indicated by the [Ca^2+^]_i_ spike occurring 3 minutes into the perturbation. At the same time, swelling is indicated by the ADC and IOS decreasing to a minimum at 5 to 7 minutes, with ADC being 3.6 ± 0.4 % reduced at 6 minutes. AXR also drops rapidly, by 21 ± 3 % at 6 minutes, but then continues to drop at a slower rate, reaching a minimum of 26 ± 5% at 14 minutes. While it appears that AXR was recovering at the end of the 20 minutes of high KCl at the end, no recovery was seen in the presence of high KCl in experiments where the duration was increased to 40 minutes (Fig S5). In the multisite exchange mechanism, AXR can decrease with ECS volume fraction due to reduced sensitivity to faster transmembrane exchange and increased sensitivity to slower geometric exchange.^28^

When washing back to normal media, [Ca^2+^]_i_ quickly starts recovering, indicating calcium being transported out of the cell likely through normal operation of the Na^+^*/*Ca^2+^ exchanger.^78^ Early in the wash the ADC and IOS decrease again prior to recovering. This may be partially because the built-up intracellular K^+^ accompanied by Cl^−^, particularly in astrocytes,^82^ takes time to be pumped out but has an immediate osmotic swelling effect. The AXR does not completely recover. With the AXR being reduced, likely the recovery of ADC and IOS is not indicating a return to the same microstructural state.

Out of the 12 experiments, from the data acquired between t=0 to 80 minutes after KCl addition, the correlation between AXR and ADC was significant in 8 cases $\left(cc=0.49\pm 0.39\right)$. The correlation between AXR and IOS was significant in all case $\left(cc=0.59\pm 0.14\right)$. The correlation between ADC and IOS was significant in nine cases $\left(cc=0.58\pm 0.21\right)$.

## Discussion

In this paper, we demonstrate simultaneous real-time NMR and optical microscopy as a tool to facilitate the translation of knowledge from cellular neurophysiology to medicine, and to validate MRI methods and their sensitivity to physiology and pathology. The temporal resolution of ADC and AXR measurements was pushed near its limit, with ADC and AXR requiring 16 seconds and 24 seconds to acquire, respectively. This is an order of magnitude faster than previous methods.^53^ Measurement variability was low enough to follow trends on timescales of minutes, sufficiently fast for changes induced by osmotic and ionic perturbations.

The static gradient (SG) spin echo (SE) diffusion NMR experiments measured diffusivity on sub-millisecond timescales. With the large 15.3 T/m gradient of the NMR MOUSE, this measurement is highly sensitive to restriction on a length scale of 800 nm.^83^ The DEXSY NMR experiment builds on this, measuring exchange between more and less restricted local environments during the prescribed mixing time. Previous studies have indicated that membranes are the sole source of restriction in the tissue^8^ Further, we found that DEXSY measurements are sensitive to transmembrane exchange, but they are also sensitive other exchange pathways, in particular geometric exchange between more and less restricted environments in the cell. We showed that with passive exchange between multiple sites, the AXR could vary drastically depending on the ECS fraction.^28^ We therefore have the working hypothesis that the AXR varies primarily with local ECS fraction. That said, we repeatedly have seen that it contains unique information distinct from the ADC.^27,29^ This study continues to advance our understanding of the determinants of changes in the ADC and AXR in live tissue, with help from complementary optical measurements.

ADC and IOS were significantly correlated in 6/8 of osmotic perturbation experiments and 9/12 of KCl perturbation experiments. Similarities in the ADC and IOS timeseries data suggest similar biophysical origins, namely cellular swelling and shrinking. The differences observed, e.g., in the kinetics, could simply be due to NMR obtaining signal from a 400 nm slice through the length of the spinal cord, compared to the microscopy signal being obtained from a small field of view. While separate accounts in the optical microscopy^65–73^ and diffusion MRI^9,27,84–88^ literature have led to general agreement that both are sensitive to cellular swelling, this is the first time that their correlation has been reported. This suggests that physiological or pathological processes previously elucidated with IOS might also be visible in vivo with diffusion MRI.

During the osmotic perturbations, AXR was only significantly correlated with ADC in 2/8 of the experiments and with IOS in 1/8 of the experiments. Interestingly, AXR was better correlated during the KCl perturbation, showing a significant correlation with ADC in 8/12 of experiments and with IOS in 12/12 of experiments. AXR remains relatively stable during and after the 100 mOsm perturbation, consistent with homeostasis being maintained, though the steady-state cell volumes vary. AXR drops during 50 mM KCl addition at the same time that [Ca^2+^]_i_ spikes and cells swell, consistent with a loss of homeostasis. AXR and [Ca^2+^]_i_ show some level of recovery during the wash from 50 mM KCl, while ADC and IOS also recover, consistent with some recovery of homeostasis. Taken together, these results suggest that AXR provides complementary information to the ADC and IOS regarding cellular homeostasis, potentially reflecting different aspects of homeostatic cell volume regulation and pathological cell swelling and recovery.

## Conclusion

While MRI and microscopy have each advanced our understanding of brain structure–function relationships, studies that apply both methods simultaneously to viable tissue remain rare. The two techniques have largely complementary strengths with limited overlap, except in their shared sensitivity to cellular-scale tissue microstructure. Here, we demonstrate the direct benefits of combining them. For the first time, we show that ADC and IOS signals tend to track one another, indicating similar sensitivity to physiological and pathological cell swelling and shrinking. As a result, the extensive IOS literature may help elucidate the mechanisms and limitations of diffusion functional imaging and guide hypotheses about diseases to which diffusion NMR may be sensitive. In contrast, AXR shows little correlation with ADC or IOS during osmolyte addition, when homeostasis is expected to be preserved, but exhibits stronger correlation during KCl addition, where we observe signs of depolarization and loss of homeostasis. This suggests that AXR is sensitive to the tissue’s homeostatic state and supports further investigation of its potential as a biomarker of tissue viability. Overall, these results illustrate how simultaneous microscopy and NMR can facilitate knowledge transfer between fields and accelerate progress at their intersection.

## Methods

### Ethics statement for animal experimentation.

All experiments were carried out in compliance with the *Eunice Kennedy Shriver* National Institute of Child Health and Human Development Animal Care and Use Committee under Animal Study Proposal (ASP) # 21–025.

Details of NMR and tissue preparation methods can be found in Refs. 8,53, and 29, and are summarized briefly here.

### Mouse spinal cord sample preparation and experimental conditions.

Spinal cords were isolated from postnatal day 1–4 Swiss Webster mice (Taconic Biosciences, Rensselaer, NY, USA) in low-calcium, high-magnesium artificial cerebrospinal fluid (aCSF) (128.35 mM NaCl, 4 mM KCl, 0.5 mM CaCl_2_, 6 mM MgSO_4_, 0.58 mM NaH_2_PO_4_, 21 mM NaHCO_3_, 30 mM D-glucose), bubbled with 95% O_2_/5% CO_2_). After dissection, cords were transferred to the NMR chamber with the ventral side up, in standard aCSF (128.35 mM NaCl, 4 mM KCl, 1.5 mM CaCl_2_, 1 mM MgSO_4_, 0.58 mM NaH_2_PO_4_, 21 mM NaHCO_3_, 30 mM D-glucose) perfused at 7 mL/min and bubbled with 95% O_2_/5% CO_2_. Perturbations were introduced by adding sucrose or KCl from a stock solution directly to the bubbled reservoir, after which the tissue was washed in an open loop using 100 mL of fresh media. Temperature was maintained at 25 ± 0.2*°*C using a fiberoptic sensor (PicoM, Opsens Solutions Inc., Québec, Canada) and a chiller-controlled water circuit.

### NMR hardware, experimental protocol, and analysis methods.

**Fig. 5.**
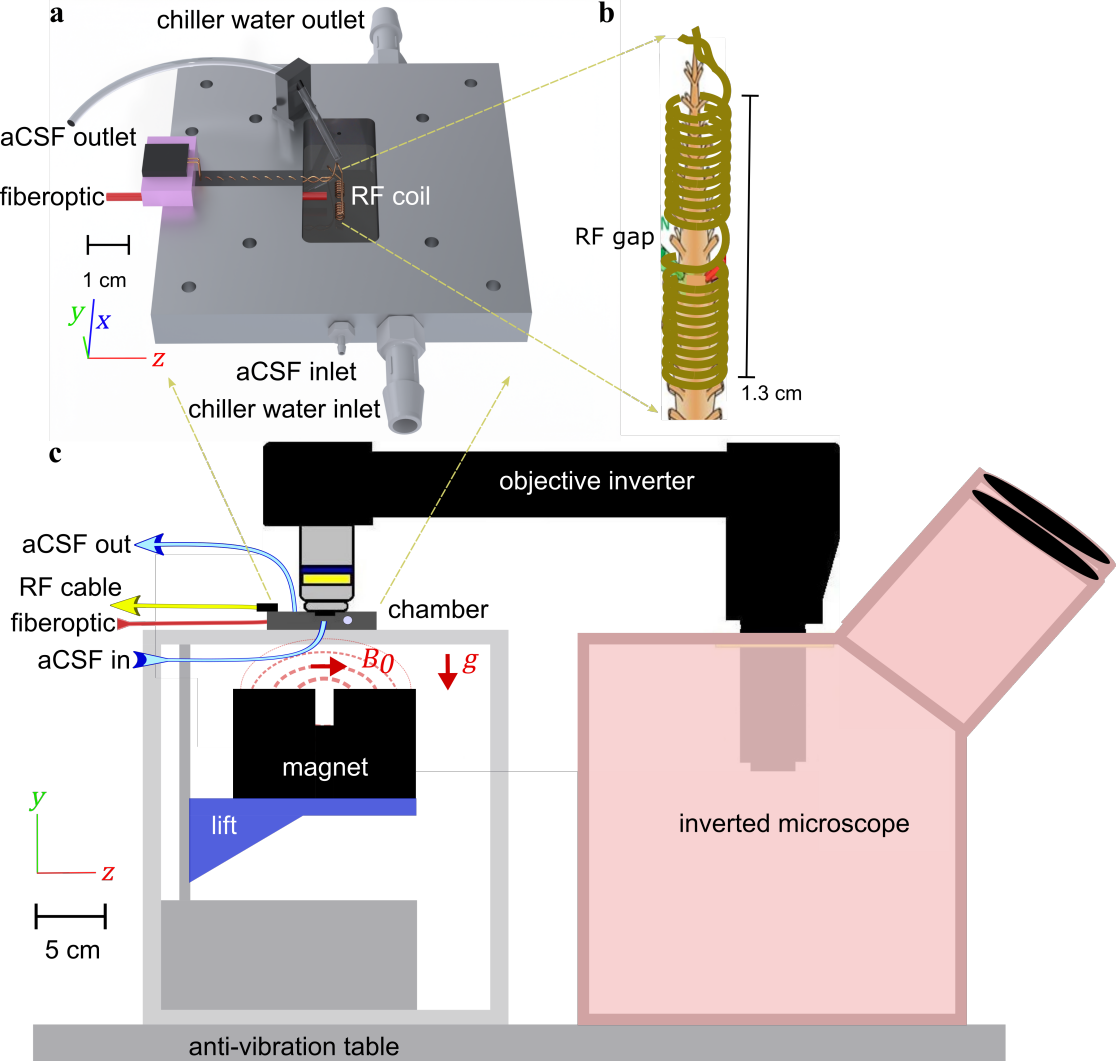
Experimental setup a) 3-D technical drawing of the test chamber. b) Drawing of the solenoid RF coil containing a mouse spinal cord showing the gap in the RF coil where the sample is imaged. c) Technical drawing of the experimental setup showing the single sided permanent magnet projecting a magnetic field on the sample from below and the inverted microscope imaging the sample from above.

NMR experiments were performed with a single-sided permanent magnet (PM10 NMR MOUSE, Magritek, Aachen, Germany) at B_0_ = 0.3239 T and gradient strength g=15.3T/m (Fig. 5).^89^ A custom test chamber and RF probe with a solenoid coil were used to maintain tissue viability while maximizing filling factor and SNR.^8,29^ The static gradient was oriented in the y-direction, perpendicular to the spinal cord, defining the slice (400μm along the cord) and diffusion encoding direction.

Sample viability was first confirmed using diffusion, Diffusion Exchange Ratio (DEXR), and $T_{1}$ recovery measurements following established protocols.^29^

Subsequently, two-point ADC and three-point DEXR measurements were performed in parallel with microscopy image acquisition. The NMR RF pulse lengths were about 2μs. NMR pulse sequences consist of an encoding block and a CPMG acquisition block.^60–62^ The acquisition block used 8000 echoes with 23.5, 25 or 28μs echo time, 4μs acquisition time, and 0.5μs dwell time and was analyzed in three ways. First, the mean angle between the real and imaginary components of the echoes yielded the phase. Second, the CPMG trains were fit with a single exponential for an $R_{2}$ estimate weighted by the encoding block. Third, the real component of echoes 5 to 8000 was summed for an SE or DEXSY signal to be used in the measurement. The two-point ADC measurement used a static gradient spin echo (SE) encoding block^58^ with b=0.025 and $2.25\;\text{ms}/\mu {\text{m}}^{2}$ (τ=0.131 and 0.587 ms) where $b=2/3\gamma^{2}g^{2}\tau^{3}$.^90^ Points acquired immediately after RF tuning were omitted due to magnetization not being at steady state. ADC was then calculated from the two SE signals using Eq. 1.

The three-point DEXR measurement used an exchange-weighted static gradient spin echo DEXSY encoding block^53^ with $\left(b_{1},b_{2}\right)=\left(2.320,\;2.170\right)\;\text{ms}/\mu {\text{m}}^{2}\left(\tau_{1},\tau_{2}=0.593,\;0.581\;\text{ms}\right)$ and mixing times $t_{m}=0.2$, 10, and 160 ms, chosen to bracket expected apparent exchange rates (AXR ~ 50–150 s^−1^). DEXSY signals were corrected for relaxation by dividing by $\text{exp}\left(-t_{m}R_{1}\right)$, where $R_{1}=1.5{\;\text{s}}^{-1}$ was obtained from standard DEXR under baseline conditions. The relaxation-adjusted signals $S_{\text{short}},S_{\text{int}}$, and $S_{\text{long}}$ were then combined to estimate the apparent exchange rate constant via Eq. 3. With 2 s repetition time, 4 scans per signal (for phase cycles) and 5 signals total, the total acquisition time was 40 seconds per ADC and AXR measurement.

### Microscopy.

An Axiovert 200 M wide-field inverted microscope (Zeiss, Germany) was equipped with an EM-CCD camera (Hamamatsu, model C9100-50) running MetaMorph (Universal Imaging) for data acquisition and experimental control. To accommodate the NMR system, the inverted microscope was placed beside the magnet and a 380 mm-long objective inverter (LSMtech, model 500) was used to place the objective over the chamber looking through a gap in the RF coil(Fig. 5). The upper arm of the microscope which carries the condenser, as well as the microscope stage were removed to allow access for connecting the objective inverter and the Rotation Immobilizer (to prevent the rotation of the nosepiece) (LSMtech). A 10x water immersion objective was used. Images were set to acquire every 30 s to be similar to the NMR acquisition rate.

The microscope interleaved measurements of the intrinsic optical signal (IOS) and intracellular calcium ([Ca^2+^]_i_) using Rhod-3 AM indicator. This used a multi LED set (Chroma Technology 89402 ET) with (Chroma Technology filters 89402X, 89402M). A multi-triggering LED illumination system (Excelitas Technologies X-cite XLED1) at 540–640nm was used for the fluorescence calcium imaging. A 10 mM stock of Rhod-3 AM dye (Thermo Fisher Scientific, Carlsbad, CA) in DMSO was diluted to 1:100 with aCSF and injected into the tissue as a bolus through a microelectrode under microscopic control. After injection, samples were let to rest for one hour prior to starting the experiment so that dye could diffuse through the tissue. For IOS imaging, transmitted near-infrared 680 nm light^72^ from an LED (Thorlab M680F4) was used. The LED was attached to a fiberoptic threaded through a hole in the side of the chamber and illuminated the sample at 90*°* to the objective. In separate experiments without NMR, the fiberoptic was placed below the chamber to illuminate the sample at 180*°* to the objective. One or more areas or ROIs on the image were delineated and the average light intensity within each ROI was determined over a time series of images. Signal intensity was then plotted as a percent change from the baseline average $\left(S-S_{0}\right)/S_{0}\times 100$.

### Statistical analysis.

All analysis was performed in MATLAB 2024b. For plotting averages (means) and standard deviations across samples, data was resampled to a 1-minute time-base and t=0 was defined as the time that sucrose or KCl was added. Correlation coefficients between NMR and microscopy timeseries were analyzed, with p≤0.01 deemed significant. One-sample t-tests were performed to determine if values were significantly different from baseline.

### 3-point method simulations and tests.

Static gradient spin echo DEXSY signals were simulated with various parameters based on those used and found experimentally, and analyzed using the 3-point method. Simulations used a model for barrier-limited two-site exchange between a free compartment and a restricted (motionally averaged) compartment^91^ with $R_{1}$ relaxation during $t_{m}$. See Supplementary Note 1 for details.

Previously-published data collected using the standard DEXR method method during OGD^29^ was downloaded from https://github.com/nathanwilliamson/WaterExchangeMeasuresActiveTransport, subsampled, and analyzed using the 3-point method. See Fig. S3 caption for details.

## Supplementary Material

1
