# Supplementary material for: Validation of diffusion and exchange imaging biomarkers via simultaneous real-time NMR and optical microscopy
Source: bioRxiv. 2025 Dec 30:2025.12.30.697003. Preprint. [Version 1] doi: 10.64898/2025.12.30.697003 (PMC12930448; doi:10.64898/2025.12.30.697003)
Supplement: 1 [file NIHPP2025.12.30.697003V1-supplement-1.pdf]

bioRxiv preprint doi: https://doi.org/10.64898/2025.12.30.697003; this version posted December 30, 2025. The copyright holder for this preprint (which was not certified by peer review) is the author/funder. This article is a US Government work. It is not subject to copyright under 17 USC 105 and is also made available for use under a CC0 license.

Supplementary Note 1: Simulation details

Static gradient spin echo DEXSY signals were simulated in MATLAB R2024a using a model for barrier-limited two-site exchange between a free compartment and a restricted compartment, ignoring spin-spin relaxation during encoding times but accounting for spin lattice relaxation (assuming the same rate R_1 for both compartments) during the mixing time t_m, using the following equation:

$$\begin{aligned} I = \Big[\left(f_e - \tfrac{f}{2}\right) \mathrm{free}(\tau_1, D_e)\, \mathrm{free}(\tau_2, D_e) + \tfrac{f}{2}\, \mathrm{free}(\tau_1, D_e)\, \mathrm{rest}(\tau_2, R) \\ + \tfrac{f}{2}\, \mathrm{rest}(\tau_1, R)\, \mathrm{free}(\tau_2, D_e) + \left(f_i - \tfrac{f}{2}\right) \mathrm{rest}(\tau_1, R)\, \mathrm{rest}(\tau_2, R) \Big] e^{-t_m R_1} \end{aligned} \quad \textbf{(S1)}$$

where τ_1 and τ_2 are the variable diffusion encoding times between the $90°$ and $180°$ pulses (i.e. half the echo time) in the first and second diffusion encoding blocks of the DEXSY sequence, D_e is the apparent diffusion coefficient in the free compartment, and f_e and f_i are the fractions of spins in the free and restricted compartment, respectively, with $f_e + f_i = 1$. Eq. S1 includes a function for attenuation due to free diffusion in the free compartment

$$\mathrm{free}(\tau, D_e) = \exp(-bD_e) \quad \textbf{(S2)}$$

with $b = \frac{2}{3}\gamma^2 g^2 \tau^3$, where γ is the gyromagnetic ratio and g is the gradient amplitude (set to $g = 15.2689$ as in the experimental study), and a function for attenuation due to restricted diffusion, i.e., motional averaging in a spherical compartment (Neuman, 1974),

$$\mathrm{rest}(\tau, R) = \exp\left(-\frac{2\gamma^2 g^2}{D} \sum_m \frac{\alpha_m^{-4}}{\alpha_m^2 R^2 - 2} \left[2\tau - \frac{3 - 4e^{-\alpha_m^2 D\tau} + e^{-2\alpha_m^2 D\tau}}{\alpha_m^2 D} \right] \right), \quad \textbf{(S3)}$$

where the first five root of $\alpha_m = \{2.0815, 5.940, 9.206, 12.405, 15.579\}/R$, R is the spherical compartment radius, and D is the free diffusion coefficient (2.15×10^{-9} m^2/s for water at $25°$C).

The exchange fraction f was defined as

$$f = (f_{ss} - f_0)\left(1 - e^{-t_m k}\right) + f_0, \quad \textbf{(S4)}$$

with

$$f_{ss} = 2 f_i f_e, \quad \textbf{(S5)}$$

where k is the exchange rate constant.

Then zero-mean Gaussian noise with signal-to-noise ratio SNR was added to the signal by sampling random numbers from a normal distribution with standard deviation $1/\mathrm{SNR}$ using $\mathrm{randn}(1,1)/\mathrm{SNR}$.

Data was generated 10000 times at each ground truth k, with random zero-mean Gaussian noise defined by the SNR added to each signal. Ground-truth k's were varied linearly over 100 values from $10 - 200\ s^{-1}$. AXR was estimated using the 3-point method. In the base-case, the AXR was not adjusted for R_1. On other cases, the AXR was adjusted for R_1 by first dividing the signals by $\exp(-R_1 t_m)$ using a prescribed R_1, which in some cases was not the true R_1. Experimentally, this is done post-hoc by utilizing prior knowledge of the apparent R_1 measured separately.

bioRxiv preprint doi: https://doi.org/10.64898/2025.12.30.697003; this version posted December 30, 2025. The copyright holder for this preprint (which was not certified by peer review) is the author/funder. This article is a US Government work. It is not subject to copyright under 17 USC 105 and is also made available for use under a CC0 license.

Supplementary figures

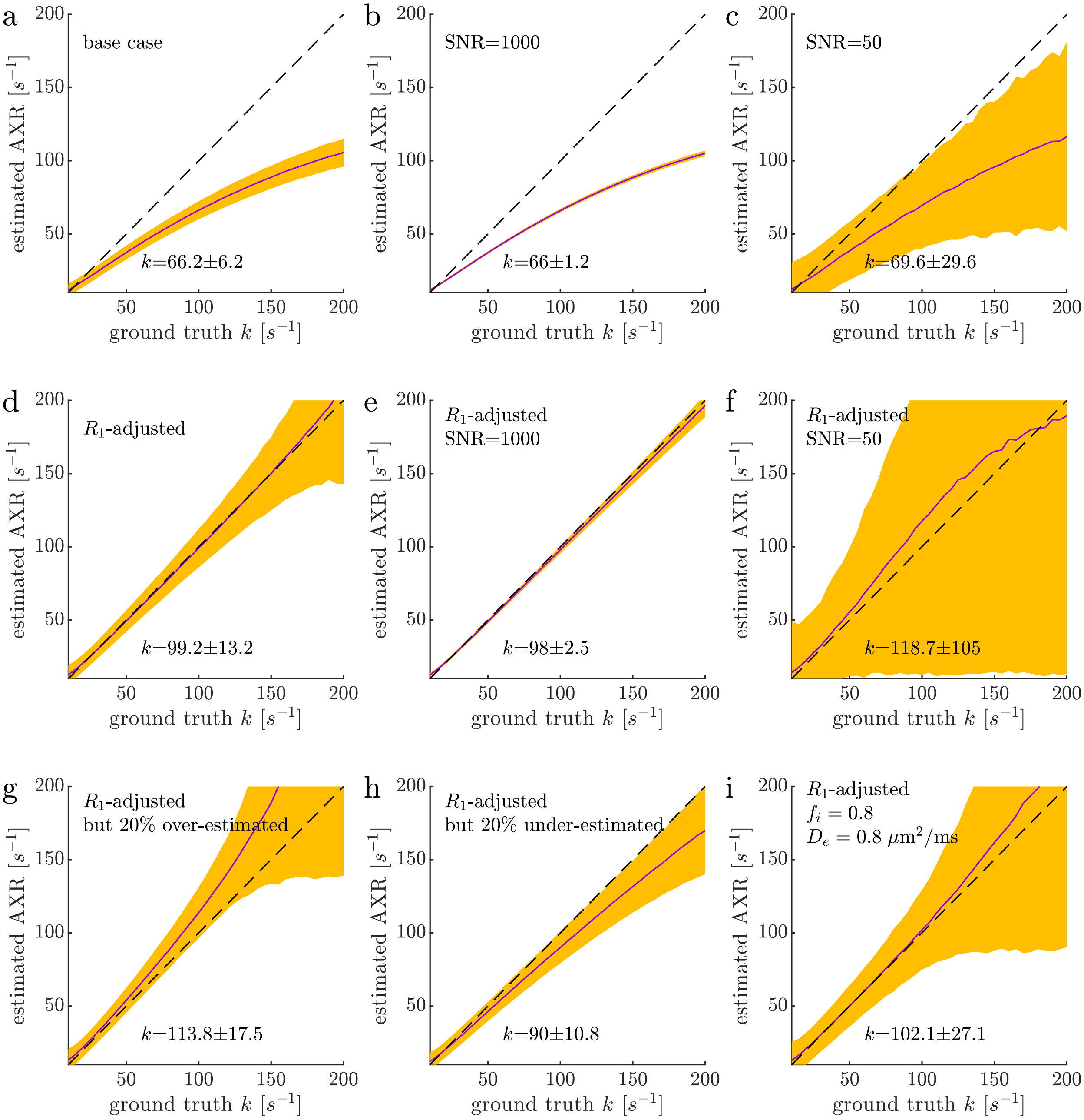

Fig. S1. Simulation tests of accuracy and precision of AXR estimates using the 3-point method, with $t_{m,\mathrm{int}} = 10$ ms. DEXSY data was simulated using Eq. S1 with radius $R = 800$ nm and a free compartment with $D_e = 1\ \mu\mathrm{m}^2/\mathrm{ms}$. Simulations used parameters $t_m = [0.2, 10, 160]$ ms and $(\tau_1, \tau_2) = (0.593, 0.58)$ ms i.e., $(b_1, b_2) = (2.320, 2.170)\ \mathrm{ms}/\mu\mathrm{m}^2$, mimicking values used experimentally (see Methods). In the "base case", $R_1 = 1.5\ s^{-1}$, $\mathrm{SNR} = 200$, and $f_i = 0.5$. Additionally, R_1 was not accounted for. In other simulations, specific parameters were varied as specified in the graphs while keeping other parameters the same as in the base case. This includes adjusting for R_1, either using the true value, or a value which was 20% over or under-estimated. The diagonal dashed line shows perfect accuracy. The solid purple line and orange band show the mean and standard deviation of AXR estimates as a function of the ground truth k. Values are displayed in each plot for the mean ± standard deviation of AXR estimates at a ground-truth $k = 100\ s^{-1}$. Standard deviations vary with SNR as expected. In a–c, Values become more and more under-estimated as the ground-truth k is increased due to the the effect of spin-lattice relaxation. in d–f, the AXR estimates were adjust for R_1 relaxation. This removes the under-estimation bias seen in a–c. g) The R_1 value used to adjust for relaxation is 20% greater than the true R_1, leading to k being over-estimated. h) The R_1 value used to adjust for relaxation is 20% less than the true R_1, leading to k being under-estimated. I) Relaxation is accounted for using the true R_1 and parameters are the same as the base case except $f_i = 0.8$ and $D_e = 0.8\ \mu\mathrm{m}^2/\mathrm{ms}$. Compared to (d), this leads to greater standard deviations but does not affect the accuracy of the measurement.

bioRxiv preprint doi: https://doi.org/10.64898/2025.12.30.697003; this version posted December 30, 2025. The copyright holder for this preprint (which was not certified by peer review) is the author/funder. This article is a US Government work. It is not subject to copyright under 17 USC 105 and is also made available for use under a CC0 license.

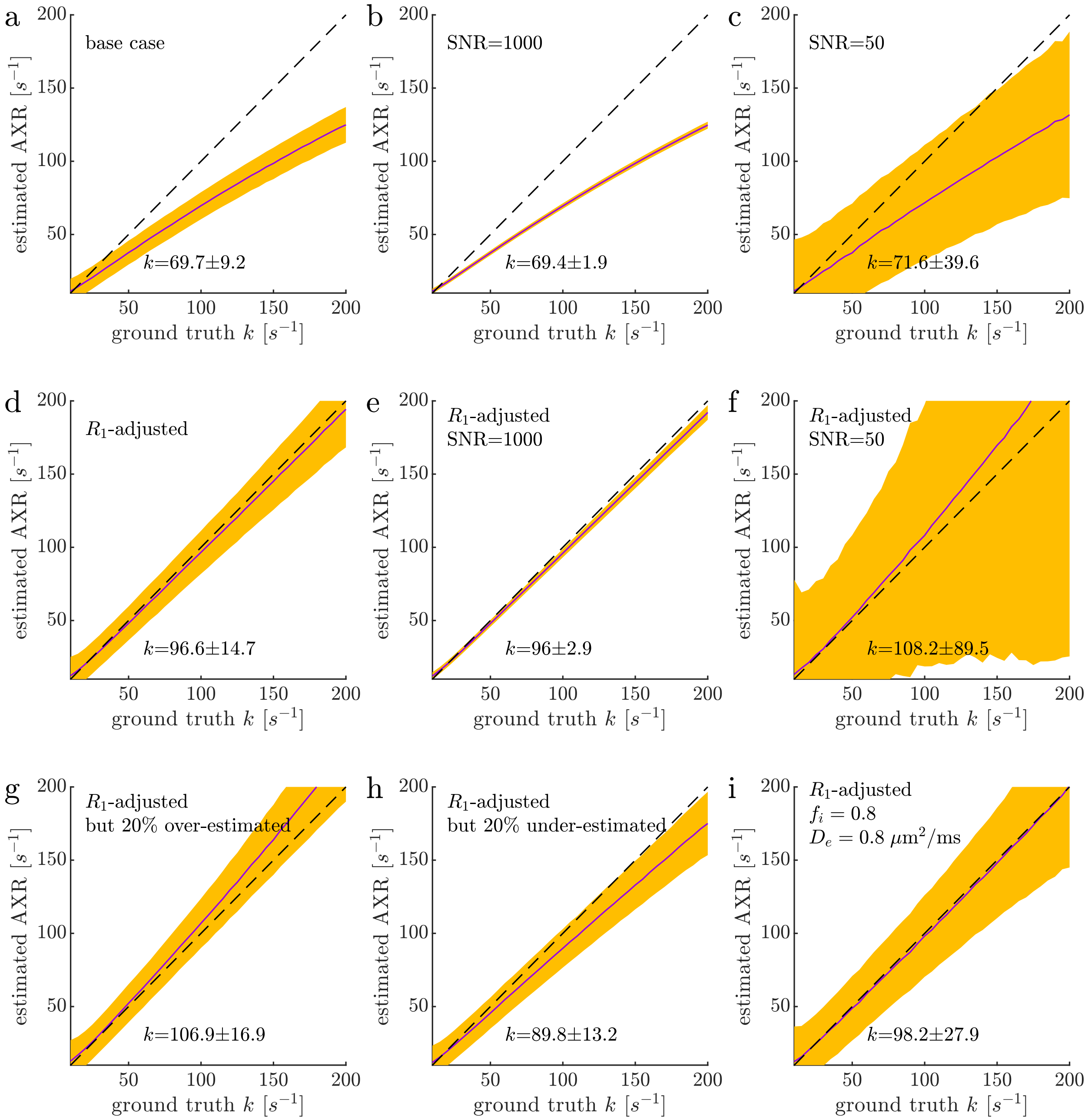

Fig. S2. Simulation tests of accuracy and precision of AXR estimates using the 3-point method, with $t_{m,\mathrm{int}} = 5$ ms All parameters are the same as in Fig. S1, except that the intermediate mixing time was set to 5 ms rather than 10 ms. This leads to reduced precision and accuracy when the ground truth $k < 100\ \mathrm{s}^{-1}$, but greater precision and accuracy for ground truth $k > 100\ \mathrm{s}^{-1}$.

bioRxiv preprint doi: https://doi.org/10.64898/2025.12.30.697003; this version posted December 30, 2025. The copyright holder for this preprint (which was not certified by peer review) is the author/funder. This article is a US Government work. It is not subject to copyright under 17 USC 105 and is also made available for use under a CC0 license.

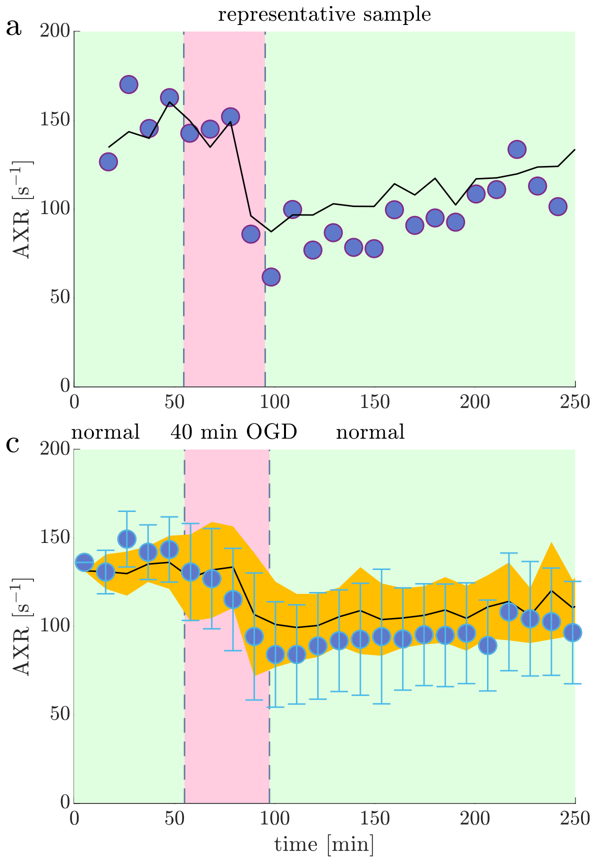

Fig. S3. Tests of 3-point analysis by subsampling OGD data. a–d) Comparisons between the 3-point AXR analysis (solid black line) and the full DEXR method (blue circles) for representative samples (a,b) and means and standard deviations (orange bands and error bars) across all samples (c,d) from previously-published (Williamson & Ravin et al., 2023) data collected during experiments involving 40 min of oxygen and glucose deprivation (OGD) (a,c, n=8), and during 70 min OGD (b,d, n=9). The full DEXR method used 22 points from $(b_1, b_2) =$ (0.089, 4.417) and (2.320, 2.170) $\mathrm{ms}/\mu\mathrm{m}^2$ with $t_m =$ [0.2, 1, 2, 4, 7, 10, 20, 40, 80, 160, 300] ms. Signal acquired with the first (b_1, b_2) pair was used to calculate the apparent R_1 and then remove it from signal acquired with the second (b_1, b_2) pair so that exchange contrast could be isolated. The 3-point analysis used the (2.320, 2.170) $\mathrm{ms}/\mu\mathrm{m}^2$ pair with $t_m =$ [0.2, 10, 160] ms and adjusted for relaxation using the effective R_1 from the full DEXR method averaged over the first 3 measurement sets in the normal condition. The 3-point analysis agrees with the full DEXR method when k values are near $150\ \mathrm{s}^{-1}$, but are biased towards higher values when k values decrease. The bias is not due to changes in relaxation because it is still present when the apparent R_1 from each set is used for adjustment. While both models assume a first order kinetic model, the 3-point method relies more heavily on this assumption because it assumes that the important timescale for exchange is captured completely by the intermediate (10 ms) encoding time. Therefore the bias may be due to multiexponential character or deviations from a first order kinetic model.

bioRxiv preprint doi: https://doi.org/10.64898/2025.12.30.697003; this version posted December 30, 2025. The copyright holder for this preprint (which was not certified by peer review) is the author/funder. This article is a US Government work. It is not subject to copyright under 17 USC 105 and is also made available for use under a CC0 license.

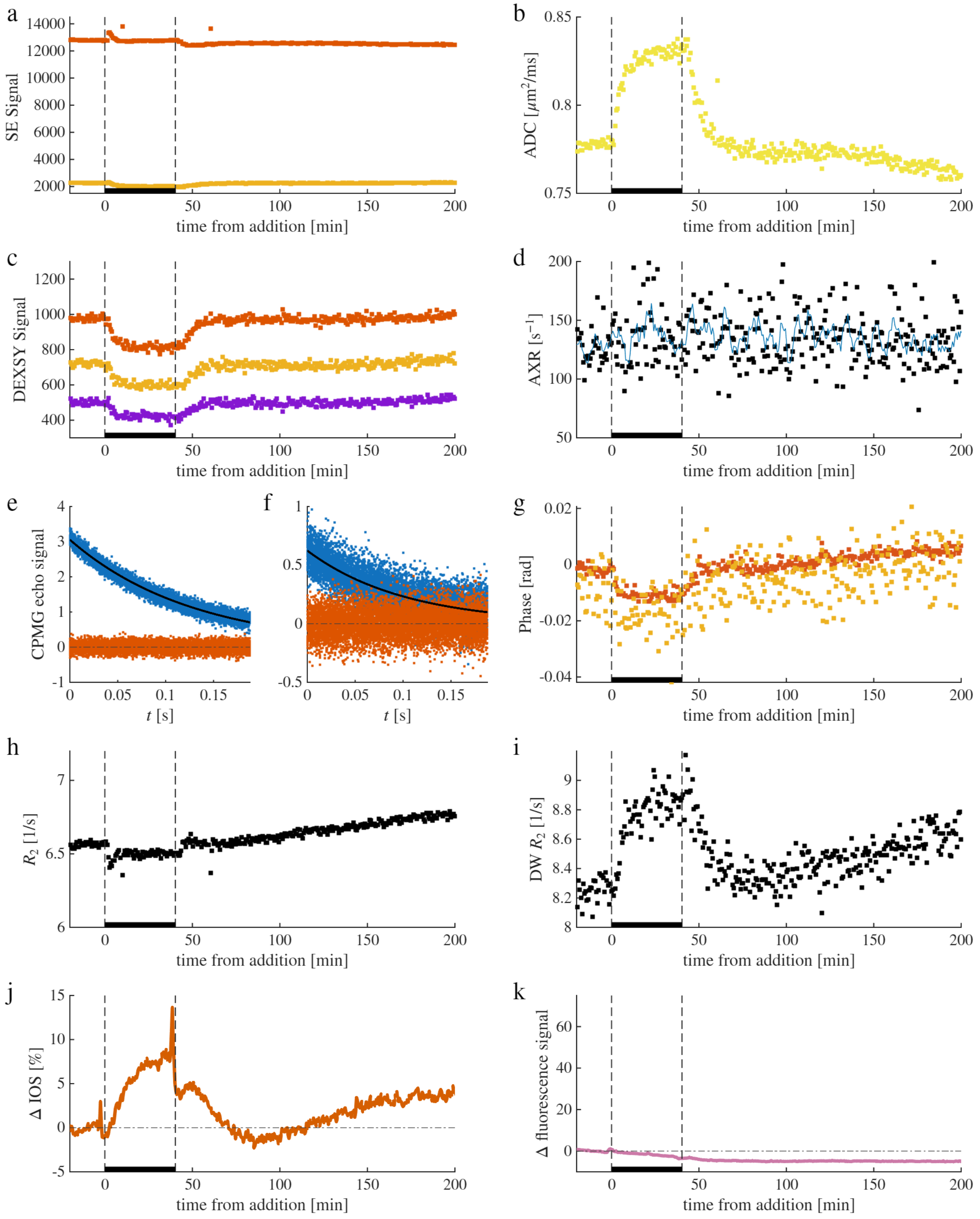

Fig. S4. Example realtime NMR and microscopy recording during an experiment involving 100 mM sucrose (osmolyte) addition and washout. Realtime recordings of two ($b = 0.025$ and $2.25\ \mathrm{ms}/\mu\mathrm{m}^2$, red and orange dots, respectively) raw spin echo diffusion signals (a), processed into one ADC measurement using Eq. 1 (b), and three ($t_m = 0.02$, 10, and 160 ms, red, orange, and blue dots, respectively) raw exchange-weighted DEXSY signals (c), processed into one AXR measurement using Eq. 2 (d). In (d), a 6-point moving average of AXR is also shown (solid blue line). (e,f) Example real (blue) and imaginary (red) components of the CPMG echo signals and exponential fits (solid black line) used to estimate R_2 acquired 18 minutes prior to KCl addition using the spin echo sequence with (e) $b = 0.025$ and (f) $2.25\ \mathrm{ms}/\mu\mathrm{m}^2$. Dot-dash line shows zero signal (g) Mean echo phase recorded the spin echo sequence with $b = 0.025$ and $2.25\ \mathrm{ms}/\mu\mathrm{m}^2$ (red and orange dots, respectively). (h,i) R_2 estimated from the CMPG decay with the spin echo echo sequence at (h) $b = 0.025$ and (i) $2.25\ \mathrm{ms}/\mu\mathrm{m}^2$. Intrinsic optical signal images (g) and fluorescence images from the channel used for recording intracellular calcium from the fluorescent indicator, Rhod-3 (h) are acquired simultaneously with the NMR, and the percent change in image intensity from a region of interest (ROI) are displayed. Here, Rhod-3 was not injected, so (k) shows the change in autofluorescence at that wavelength. The lack of change indicates that autofluorescence does not confound intracellular calcium imaging. Spuriously higher signals (a) and lower R_2 (h) at roughly 10 and 60 min show when tuning was performed.

bioRxiv preprint doi: https://doi.org/10.64898/2025.12.30.697003; this version posted December 30, 2025. The copyright holder for this preprint (which was not certified by peer review) is the author/funder. This article is a US Government work. It is not subject to copyright under 17 USC 105 and is also made available for use under a CC0 license.

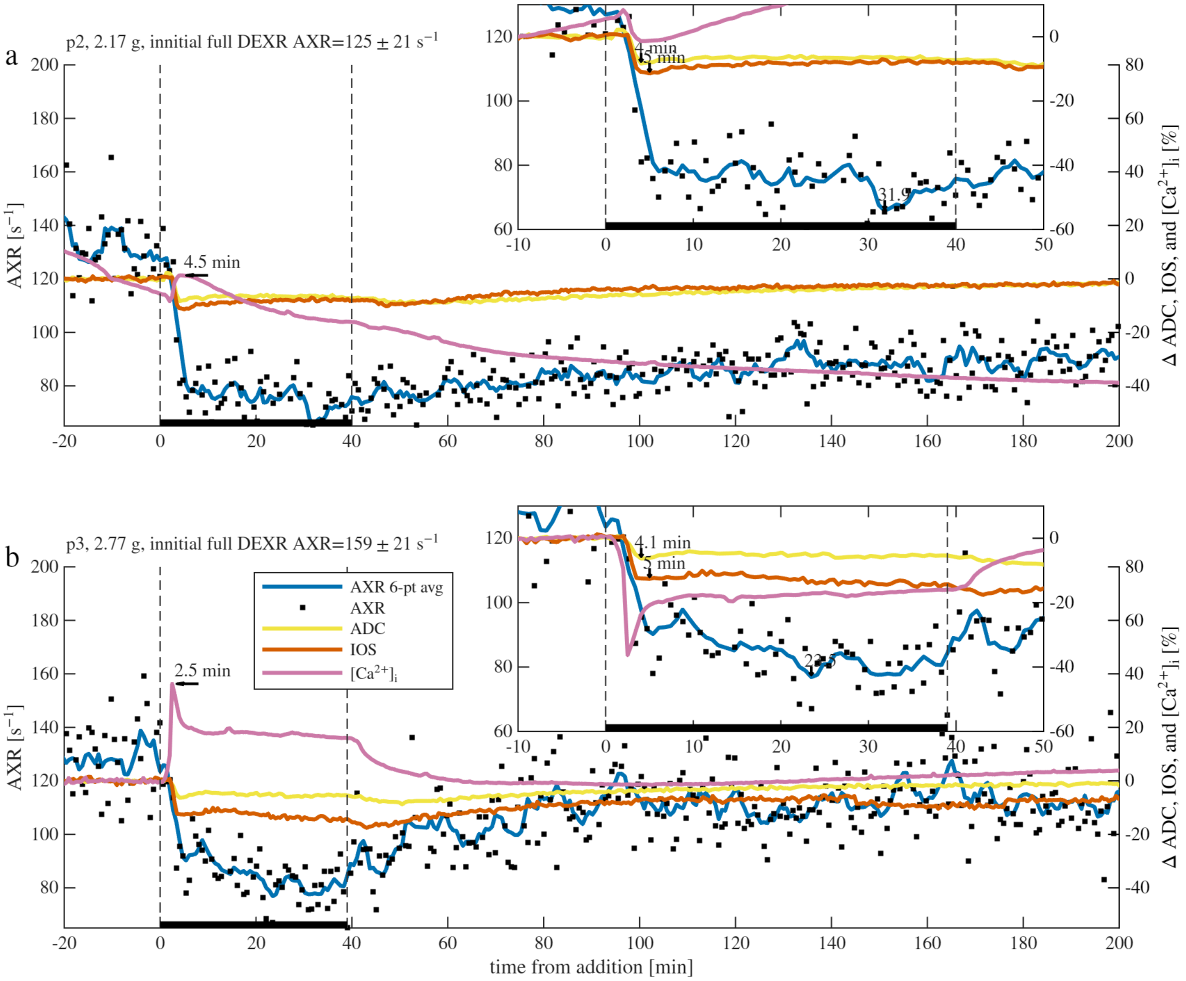

Fig. S5. Individual recordings of simultaneous realtime hydrophysiology NMR and microscopy showing cellular shrinkage caused by 40 minutes of 50 mM KCl. a-d) Individual recordings from $n = 2$ samples. A 6-point moving average of AXR is also shown (solid blue line). Correlations were deemed significant when p-values were less than 0.01 and not significant (NS) otherwise. Correlation coefficients (and p-values) between AXR and ADC, AXR and IOS, AXR and $[\mathrm{Ca}^{2+}]_\mathrm{i}$, ADC and IOS, ADC and $[\mathrm{Ca}^{2+}]_\mathrm{i}$, and IOS and $[\mathrm{Ca}^{2+}]_\mathrm{i}$ from data acquired between t=0 to 80 minutes for each recording are

[a) 0.77, 0.74, NS, 0.71, 0.50, NS ($p \ll 0.001,\ \ll 0.001,\ = 0.02,\ \ll 0.001,\ \ll 0.001,\ 0.08$)],

[b) 0.32, 0.69, -0.67, 0.85, NS, NS ($p = 0.003,\ \ll 0.001,\ \ll 0.001,\ \ll 0.001,\ = 0.08,\ = 0.05$)].
